# Brain Atrophy as a Measure of Neuroprotective Drug Effects in Multiple Sclerosis: Influence of Inflammation

**DOI:** 10.3389/fnhum.2016.00226

**Published:** 2016-05-13

**Authors:** Tatiana Koudriavtseva, Caterina Mainero

**Affiliations:** ^1^Multiple Sclerosis Clinical and Research Unit, Department of Systems Medicine, University of Rome Tor VergataRome, Italy; ^2^Athinoula A. Martinos Center for Biomedical Imaging, Massachusetts General HospitalBoston, MA, USA; ^3^Department of Radiology, Harvard Medical SchoolBoston, MA, USA

**Keywords:** multiple sclerosis, brain atrophy, inflammation, neuroprotection, disease modifying therapy

Multiple Sclerosis (MS) is a chronic inflammatory and neurodegenerative disease of the central nervous system, and the second cause of neurological disability after trauma in young adults in the Western world. The efficacy of disease-modifying treatments (DMTs) in relapsing-remitting (RR) and secondary-progressive (SP) MS is usually surrogated by assessing new/enlarged T2 and gadolinium (Gd)-enhancing lesions on magnetic resonance imaging (MRI) scans. More recently, brain atrophy has been incorporated as an outcome measure in MS clinical trials due to its reproducibility, correlation with disability, and detectability from early disease stages. Brain atrophy measures, however, could lead to equivocal conclusions if a series of factors are not taken into account, particularly the influence of inflammation.

Some clinical trials evaluating the effects of the same drugs on brain atrophy reported conflicting results between them, which have been mainly attributed to the inclusion of heterogenous patient populations, and/or the use of different MRI acquisitions and post-processing methods (De Stefano et al., 2014). A recent systematic review and meta-analysis of 12 studies evaluating the effects of first-generation DMTs on annualized percentage brain volume change (PBVC) over at least 12-month follow-up by Structural Image Evaluation of Normalized Atrophy algorithm reported that its pooled value was similar between treated and untreated patients (−0.69 and −0.71%, respectively; Vollmer et al., 2015). Another meta-analysis, which included four large studies involving RRMS cases, showed only modest beneficial effects of DMTs on PBVC (Tsivgoulis et al., 2015). Drug effects were greater during the second year than the first year of therapy and linearly increased with therapy prolongation, with no heterogeneity across trials using different DMTs or various neuroimaging protocols (Tsivgoulis et al., 2015).

Here, we discuss the potential factors related to the presence of inflammation that could influence brain volume evolution in MS, both in the short- and long-term, and explain, at least in part, the conflicting results on the neuroprotective effects of DMTs.

First, the “pseudoatrophy” phenomenon must be taken into account when brain volume changes are used to evaluate DMTs' neuroprotective effects in MS clinical trials. This phenomenon, which occurs during the first 6 months up to a year from beginning of therapy, is due to resolution of the on-going inflammation, and is predominant for DMTs with strong anti-inflammatory properties. Due to the “pseudoatrophy” phenomenon, DMTs' positive effects on brain atrophy compared with placebo are usually more evident in the second year of therapy (De Stefano et al., 2014). As “pseudoatrophy” is believed to reflect fluid shifts and resolution of oedema rather than true tissue loss, this short-term phenomenon can lead to misinterpretation of brain atrophy results if the baseline brain volume coincides with treatment onset. Interestingly, different DMTs exhibit “pseudoatrophy” effects of variable entity, though in all cases the effect is prevalent in MS patients with evidence of active inflammation at baseline. For example, in clinical trials evaluating the efficacy of fingolimod, evidence of “pseudoatrophy” was found only in the subgroup of patients who showed active inflammation at baseline (Radue et al., 2015). As there is still much to learn about “pseudoatrophy” and its dynamics, it might be appropriate to state that within the first 6 months of starting DMTs, early changes of brain volume are unlikely to reflect true tissue loss and should be interpreted with caution. In light of this, it seems appropriate that for assessing brain atrophy changes under DMTs, the baseline brain volume should be calculated from MRI scans acquired at 6 months after the beginning of therapy.

It has been suggested that gray matter (GM) volume loss in MS could be a more sensitive indicator of disease progression than white matter (WM) atrophy, as “pseudoatrophy” effects are greater in WM due to its larger inflammatory activity, which is accompanied by blood-brain barrier breakdown, local fluid leakage, and increased brain perfusion (Koudriavtseva et al., 2015). These processes are less evident in GM, which is usually characterized by decreased vascular perfusion. Furthermore, GM atrophy rate increases as disease progresses, whereas WM changes tend to remain substantially similar across disease stages (De Stefano et al., 2014). Indeed, a recent study re-evaluating the effect of interferon-beta-1a on GM and WM fractions over 2 years showed that GM change is the most accurate index of tissue loss and disability progression, although the study used 5 mm thick slices, which might not be adequate for measuring reliably GM atrophy (Fisher et al., 2016).

In SPMS, a disease stage predominantly characterized by GM neurodegenerative pathological processes, assessment of GM volume loss would be the most appropriate tool for evaluating neuroprotective drug effects (Geurts et al., 2009). Furthermore, it might be worthwhile assessing the GM atrophy in past DMTs trials in RRMS (Fisher et al., 2016) to reduce potential “pseudoatrophy” confounding effects that prevail in the WM. This could allow to elucidate whether the neuroprotective effects of some DMTs in RRMS are greater than originally thought (Tsivgoulis et al., 2015; Vollmer et al., 2015). Methods for standardizing GM atrophy assessments, however, are still lacking, and the imaging resolution used in clinical trials is usually not adequate for accurate GM volume measurements, especially in the cortex.

Finally, another aspect that could account for the limitations for using brain atrophy measures in DMTs clinical trials in MS derives from the complex relationship between the degree and extent of inflammatory activity and brain volume changes, which seems to extend far beyond the short-term pseudoatrophy phenomenon, regardless of therapy. For example, the effects of baseline brain inflammatory activity in RRMS represented by MRI gadolinium enhancement at the onset of natalizumab therapy resulted in greater brain tissue loss compared to cases with no evidence of MRI baseline inflammation at 12- and 24-month follow-up but not at 36-month follow-up (Sastre-Garriga et al., 2015). Similarly, a previous work investigating 36-month interferon-beta effect on brain atrophy in SPMS showed that drug effects as compared to placebo were detected only in patients with no baseline Gd-enhancing lesions, whereas there was a trend toward a greater atrophy in treated patients compared to placebo patients having at least one baseline Gd-enhancing lesion (Molyneux et al., 2000). Furthermore, the rate of brain atrophy over 2 years in three pivotal phases in three studies evaluating the efficacy of fingolimod therapy in RRMS was found to be influenced by both baseline and on-study disease activity (Radue et al., 2015). Specifically, both high baseline T2 lesion volume and Gd-enhancing lesion count were identified as the best predictors of greater PBVC, whereas high T1-hypointense lesion and low normalized brain volumes were weaker negative baseline predictors. These findings suggest that whole-brain atrophy over the 2-year follow-up is influenced mainly by recent and, to a lesser extent, by remote disease activity (Radue et al., 2015). Interestingly, in one of these studies (placebo-controlled trial of oral fingolimod FREEDOMS), the PBVC was also predicted by the number of relapses occurring in both 1- and 2-year periods preceding the study (Radue et al., 2015). Despite the fact that fingolimod reduced the annual brain atrophy rate by approximately one-third compared with other trial groups, an influence of baseline disease activity was reported not only in the treated group (fingolimod, interferon-beta-1a) but also in the placebo group, thereby indicating its conditioning on brain atrophy regardless of therapy. On-study disease activity including cumulative number of Gd-enhancing lesions, number of both T2 new/enlarged lesions, and confirmed relapses were also correlated with the PBVC (Radue et al., 2015).

These findings strongly support for including baseline T2 lesion load, Gd-enhancing lesion count, and number of previous relapses in both randomization and statistical modeling of clinical trials in MS that include brain atrophy as endpoint. A recent MS-STAT trial reported a protective effect of simvastatin on brain atrophy and disability in SPMS (Chataway et al., 2014). In this study, however, the placebo group compared with the simvastatin-treated one had, apparently, more active disease by means of greater number of relapses in the previous 12 and 24 months, and greater on-study rate of T2 new/enlarging lesions. Unfortunately, in this study, baseline T2 lesion load and Gd-enhancing lesion count were not reported. These limitations may, thus, leave some reservations about the protective effects of simvastatin on neurodegeneration, as they simply could reflect the consequence of less baseline and on-study disease activity in the treated group.

Indeed, MS patients with higher levels of disease activity (expressed by either the presence of Gd-enhancing lesions, new T2 lesions, and/or clinical relapses) tend to have greater rates of brain atrophy, and this is a consistent finding across different trials, treatment arms, and disease subtypes. This correlation is likely related to the dynamics of MS pathologic processes: inflammation determines demyelination that likely leads to neuroaxonal degeneration and tissue loss, which in turn is reflected by brain volume loss on MRI. Hence, for the most part, the neuroprotective effects of DMTs in MS might be secondary to a reduction of inflammatory damage and, therefore, delayed in time.

In conclusion, the presence of on-going inflammatory activity leads to a fluctuation in whole brain, and mostly in WM volume, due to both fluid and inflammatory infiltrates. Paradoxically, alleviation of this inflammation causes a pronounced atrophy in the first treatment period (so-called “pseudoatrophy”). In the longer term, inflammation might bring to greater demyelination and neuroaxonal degeneration, leading to larger brain tissue loss. This suggests that the neuroprotective effects of most DMTs might be largely secondary to their anti-inflammatory effects with delayed reduction of atrophy rate, and this needs to be accounted for when evaluating potential neuroprotective effects of these therapies.

## Author contributions

All authors listed, have made substantial, direct and intellectual contribution to the work, and approved it for publication.

### Conflict of interest statement

TK reports consulting fees from Bayer Schering, and Institutional grant from Merck Serono, Biogen Idec, Novartis, Bayer Schering outside the submitted work. CM reports speaker fees from Biogen, and research support from Merck Serono outside the submitted work. The reviewer JE and handling Editor declared their shared affiliation, and the handling Editor states that the process nevertheless met the standards of a fair and objective review.
